# Entropy and Mixing Entropy for Weakly Nonlinear Mechanical Vibrating Systems

**DOI:** 10.3390/e21050536

**Published:** 2019-05-26

**Authors:** Zahra Sotoudeh

**Affiliations:** Department of Engineering, California State Polytechnic University, Pomona, CA 91768, USA; zsotoudeh@cpp.edu

**Keywords:** entropy, vibration, the second law of thermodynamics, Duffing oscillator, Henon–Heiles oscillators, weakly nonlinear structures

## Abstract

In this paper, we examine Khinchin’s entropy for two weakly nonlinear systems of oscillators. We study a system of coupled Duffing oscillators and a set of Henon–Heiles oscillators. It is shown that the general method of deriving the Khinchin’s entropy for linear systems can be modified to account for weak nonlinearities. Nonlinearities are modeled as nonlinear springs. To calculate the Khinchin’s entropy, one needs to obtain an analytical expression of the system’s phase volume. We use a perturbation method to do so, and verify the results against the numerical calculation of the phase volume. It is shown that such an approach is valid for weakly nonlinear systems. In an extension of the author’s previous work for linear systems, a mixing entropy is defined for these two oscillators. The mixing entropy is the result of the generation of entropy when two systems are combined to create a complex system. It is illustrated that mixing entropy is always non-negative. The mixing entropy provides insight into the energy behavior of each system. The limitation of statistical energy analysis motivates this study. Using the thermodynamic relationship of temperature and entropy, and Khinchin’s entropy, one can define a vibrational temperature. Vibrational temperature can be used to derive the power flow proportionality, which is the backbone of the statistical energy analysis. Although this paper is motivated by statistical energy analysis application, it is not devoted to the statistical energy analysis of nonlinear systems.

## 1. Introduction

Statistical energy analysis (SEA) is a method of predicting energy transmission in systems that are undergoing high-frequency vibration. SEA is a computationally inexpensive reduced order model that serves as an alternative to numerical approaches such as finite element analysis [1]. In classical SEA, a power flow proportionality law is used to relate the ensemble average energies of individual structural or acoustic subsystems to the ensemble average energy flow rate between subsystems [1,2,3,4,5,6]. These methods are based on the first law of thermodynamics, i.e., the principle of conservation of energy. This principle is used to obtain the energy balance equations of the systems [7]. In SEA, energy is assumed to flow from systems with higher average modal energies to systems with lower average modal energies [8].

The classical SEA approach is valid under a number of limiting assumptions [1,8,9,10,11]. Efforts to examine SEA for nonlinear systems have been limited in scope [12]. To the best of our knowledge, there are not many papers devoted to nonlinear SEA. It has been accepted that SEA is used for linear systems. Ref. [13] studies the coupling between a linear acoustic fluid and a nonlinear structure. It shows that although the steady-state response of the structure strongly depends on the structural nonlinearities, the basic power proportionality law of SEA is preserved. Ref. [12] studies the energy cascade phenomenon due to large deformations in coupled vibrating plates. Ref. [12] suggests defining subsystems based on modal analysis instead of physical subsystems. The response of the structure in the frequency domain is divided into frequency bands, and the energy flow between the frequency bands is calculated. Although in this paper we do not study the dynamic behavior of a nonlinear system, we are developing the mathematical requirement for expanding SEA for studying nonlinear systems.

The concept of entropy has been introduced for mechanical oscillators [7,14,15,16,17] in order to improve SEA. Entropy approaches do not assume linearity, and consequently can be applied to nonlinear systems. Using the entropy concept for vibrating structures may result in an alternative way of deriving the power flow law for nonlinear systems. This is expected to provide insight into restrictive assumptions of SEA and possibly expand the applicability of it.

Carcaterra has introduced Khinchin’s entropy [7,18] for weakly nonlinear systems with (1) dead-zone nonlinearity and (2) polynomial anharmonic potentials [15]. However, the accuracy of this approach has yet to be investigated numerically for nonlinear systems [7,15,19]. This paper further expands this approach by introducing complete derivation of entropy using the perturbation method for Duffing oscillators and Henon–Heiles oscillators. We also propose verifying our calculation of phase volume using perturbation methods against numerical computation.

This paper extends the author’s previous work on linear systems [17] to the nonlinear regime. Ref. [17] introduces mixing entropy for linear systems. It includes a mathematical proof showing mixing entropy is always non-negative and illustrates this concept for several numerical examples. To determine Khinchin’s entropy, the equi-energy volume enclosed by a system’s trajectory in phase space must be known analytically. For linear oscillators, this phase volume is a simple ellipsoid [7,17]. This paper will demonstrate that this is not the case for nonlinear systems. Additionally, it is expected that entropy satisfies the entropy inequality property [7] for nonlinear systems. The entropy inequality property ensures that the entropy of a system must not decrease as a result of coupling with other systems. This is shown by introducing the concept of a mixing entropy for nonlinear systems. The mixing entropy must be strictly non-negative for the second law of thermodynamics to be satisfied in the context of mechanical vibrating systems.

Although there are several definitions of entropy, [20,21], papers motivated by use of entropy in SEA have all used Khinchin’s entropy, for example Refs. [7,14,15,22]. We explored three definitions of entropy for SEA applications in Ref. [23] and concluded Khinchin’s entropy is the most useful definition for SEA applications. Therefore in this paper, we use Khinchin’s entropy.

The contributions of this paper are three-fold: (1) conducting a detailed examination of entropy for two nonlinear vibrating systems, (2) introducing mixing entropy for nonlinear vibrating systems, and (3) deriving vibroacoustic temperatures for nonlinear systems to be used in the context of SEA. In this paper, entropy is examined in detail for two nonlinear two-degree-of-freedom (2-DOF) systems. The first system consists of two Duffing oscillators [15,21] coupled by a linear spring. This system is always stable. The second system is a set of Henon–Heiles oscillators [21,24]. The Henon–Heiles system contains a repulsive anharmonic potential, which causes instability if one of the oscillators has sufficient energy to escape from the origin.

## 2. Motivation

The foundational equation of SEA is the power flow proportionality law, which states that the average power flow (energy exchange rate) between two subsystems is proportional to the difference between the average modal energies of the two subsystems:

(1) $$P_{12}\propto \frac{E_{1}}{N_{1}}-\frac{E_{2}}{N_{2}},$$

where $P_{12}$ is the power flow between the subsystems, and $E_{1}/N_{1}$ and $E_{2}/N_{2}$ are the average modal energies of the subsystems. Equation (1) is a consequence of the first law of thermodynamics, and is a special case of a fundamental energy relationship [14]:

(2) $$P_{12}\propto T_{1}-T_{2},$$

where $T_{1}$ and $T_{2}$ denote the vibroacoustic temperatures of the first and second subsystems, respectively. Equation (2) states that the power flow between mechanically vibrating systems is proportional to the difference between their vibroacoustic temperatures. It is understood that the entropy concept can be used to obtain relationships between energy and vibroacoustic temperature. Carcaterra has used entropy to show that T=E/N gives the vibroacoustic temperature for linear systems [15]. Since entropy approaches make no assumptions about system linearity, they can be applied to nonlinear systems. Therefore, it is expected that entropy analyses can be used to obtain SEA power flow relationships for nonlinear vibroacoustic systems. This expectation is the main motivation for the work presented in this paper. In this paper we show that although the calculation is more complex, perturbation method can be used to obtain the temperatures for weakly nonlinear vibrating systems.

With the goal of weakening the constraints of SEA, entropy has also been examined in the context of strongly coupled oscillators [25] and nonconservative oscillators [26]. This paper is an extension of the author’s previous work to nonlinear systems. By examining entropy for nonlinear systems, vibroacoustic temperatures can be obtained which will enable the development of SEA power flow relationships for nonlinear vibroacoustic systems.

## 3. Overview of Khinchin’s Entropy

Consider a mechanical system, *S*, that is described by a set of *N* positions, $q_{i}$, and *N* generalized velocities, $p_{i}=\dot{q_{i}}$, where i=1,2,…,N. The micro-state of *S* is defined as the set of $\left\{q,\dot{q}\right\}$. The macro-state of *S* is given by its total energy, which is a global function of the micro-state [7]. Assume we can describe this system with Hamiltonian equations:

(3) $$\begin{array}{cc} \frac{\partial H}{\partial p_{i}} & ={\dot{q}}_{i}\\ \frac{\partial H}{\partial q_{i}} & ={\dot{p}}_{i}. \end{array}$$

There are infinitely many possible micro-states for a given macro-state for each system. Thus, knowledge about the exact system configuration is lost when only the macro-state of the system is known. The main goal of SEA is to estimate the energies of systems and the subsystems without knowing the exact configuration, $\left\{q,\dot{q}\right\}$. The concepts of micro- and macro-states play an important role when defining entropy [7,15].

Khinchin’s definition of entropy [18] has been introduced for vibrating mechanical systems [7,15,17]. A measure of the number of possible micro-states of a system should be defined to obtain Khinchin’s entropy. Consider a system, *S*, described by *N* position and velocity pairs. In 2N-dimensional phase space, the trajectory of this system sweeps out a closed equi-energy surface over time. There are infinite possible micro-states that fall on this trajectory. The volume, *V*, enclosed by a system’s closed-surface trajectory in phase space is called phase volume. The derivative of the phase volume, *V*, with respect to energy is called a structure function, Ω. Both phase volume, *V*, and the structure function, Ω are measure numbers of possible micro-states of the system.

(4) $$\Omega \left(E\right):=V^{\prime }\left(E\right),$$

where the energy, *E*, is the total mechanical energy of the system, the summation of potential and kinetic energy. The structure function is useful because it is an invariant measure [7,21]. Taking the Laplace transform of the structure function yields the generating function of the system,

(5) Φ(s):=L{Ω(E)}.

The generating function is central towards defining Khinchin’s entropy. The generating function corresponding to *S* is given by the product of the generating functions of its subsystems, assuming the total energy is the sum of subsystems’ energies. Such subsystems are referred to as non-overlapping subsystems [7,17]. Denoting the generating function of each subsystem as $\Phi_{i}$, the generating function of the entire system is

(6) $$\Phi \left(s\right)=\prod_{i=1}^{N}\Phi_{i}\left(s\right).$$

Equation (6) is the law of composition of the generating function for a system composed of *N* non-overlapping subsystems [7,17]. The law of composition is a useful tool for determining the generating functions of weakly coupled systems. This law is proved in Ref. [7].

Khinchin’s entropy is defined as [18]

(7) $$\begin{array}{c} H\left(E,\sigma \right):=\ln[e^{\sigma E}\Phi \left(\sigma \right)]+C, \end{array}$$

Subject to

(8) $$\frac{\Phi^{\prime }\left(\sigma \right)}{\Phi (\sigma )}=-E,$$

where *E* is the energy of the system, *C* is an arbitrary scaling constant, and Φ(σ) is the generating function with the argument s=σ. This condition, Equation (8), ensures that *H* is minimized at s=σ.

The entropy of a system must equal the sum of the entropies of its constituent subsystems.

(9) $$H\left(\Phi \left(s\right)\right)=H\left(\prod_{i=1}^{N}\Phi_{i}\left(s\right)\right)=\sum_{i=1}^{N}H_{i}\left(\Phi_{i}\left(s\right)\right).$$

Khinchin’s entropy satisfies this additive entropy property [7].

One can derive an expression for Khinchin’s entropy of a system by following these steps: [7]

1. Calculating the equi-energy volume enclosed by the system’s closed-surface trajectory in phase space, *V*.
2. Determining the structure function, Ω.
3. Determining the generating function, Φ.
4. Finding the value s=σ that satisfies Equation (8).
5. Substituting the generating function and σ into Equation (7).

This procedure does not make assumptions about the linearity of the system, and is not limited in this respect [7]. As long as the equi-energy volume can be determined, the remaining steps of the procedure are standard. For linear systems, the equi-energy volume is an ellipsoid, and can be determined analytically [7,25,27]. However, this is not generally the case for nonlinear systems.

### 3.1. Linear Systems

For an *N*-degree-of-freedom (*N*-DOF) linear conservative system, the energy of the system can be expressed in terms of its modal coordinates, $q_{i}$ and ${\dot{q}}_{i}$:

(10) $$\sum_{i=1}^{N}a_{i}q_{i}^{2}+\sum_{i=1}^{N}b_{i}{\dot{q}}_{i}^{2}=E,$$

where $a_{i}$ and $b_{i}$ are constants that depend on the modal stiffnesses and masses. Dividing Equation (10) by *E* yields

(11) $$\sum_{i=1}^{N}\lambda_{i}q_{i}^{2}+\sum_{i=1+N}^{2N}\lambda_{i}{\dot{q}}_{{}_{i-N}}^{2}=1,$$

where $\lambda_{i}=a_{i}/E$ for i=1,2,…N and $\lambda_{i}=b_{i}/E$ for i=1+N,2+N,…2N. Expressing Equation (11) in matrix form [25],

(12) $$q^{T}\Lambda q=1,$$

where Λ is a symmetric coefficient matrix defined as

(13) $$\Lambda =\left[\begin{array}{cccc} \lambda_{1} & 0 & 0 & 0\\ 0 & \lambda_{2} & 0 & 0\\ 0 & 0 & \ddots & 0\\ 0 & 0 & 0 & \lambda_{2N} \end{array}\right],$$

and the coordinate matrix $q={\left\{q_{1},\cdots ,q_{N},{\dot{q}}_{1},\cdots ,{\dot{q}}_{N}\right\}}^{T}$. From here, the $i^{th}$ semi-axis of the phase volume is

(14) $$c_{i}=\sqrt{\frac{1}{\lambda_{i}}}.$$

The volume of an ellipsoid is the product of the semi-axes, multiplied by a constant. Therefore,

(15) $$V\left(E\right)\propto \prod_{i=1}^{2N}\sqrt{\frac{1}{\lambda_{i}}}\propto E^{N}.$$

Thus, the volume enclosed by the phase-space trajectory of an *N*-DOF conservative linear system is

(16) $$V\left(E\right)=BE^{N},$$

where *B* is a constant that depends upon the physical parameters of the system, such as the stiffness and modal mass. The structure function, defined in Equation (4), is

(17) $$\Omega \left(E\right)=NBE^{N-1},$$

which yields the generating function

(18) $$\Phi \left(s\right)=N!Bs^{-N}.$$

Substituting Equation (18) into Equation (8) and solving for s=σ returns σ=N/E. Substituting σ into Equation (18) yields the generating function corresponding to the volume in Equation (16):

(19) $$\Phi \left(E\right)=N!B{\left(\frac{E}{N}\right)}^{N}.$$

Then, substituting Equation (19) into Equation (7) and expanding the result yields

(20) $$\begin{array}{cc} H_{L}\left(E,N\right) & =N\ln\frac{E}{N}+N+\ln N!+\ln B+C\\ & =N(1+\ln\frac{E}{N})+\ln N!+\ln B+C, \end{array}$$

where $H_{L}$ is the entropy of an *N*-DOF linear system. Since the constant *C* is arbitrary, it can be defined such that [22]

(21) $$H_{L}\left(E,N\right)=N\left(1+\ln\frac{E}{N}\right).$$

According to the thermodynamic definition of entropy, the temperature of the system is given by $T={\left(dH/dE\right)}^{-1}=1/\sigma$, where σ is the value that satisfies Equation (8). For the entropy given in Equation (21), this yields T=E/N. The parameter T=E/N gives the average modal energy of the system [15], which is consistent with the vibroacoustic temperatures used in the power flow proportionality of SEA [14].

### 3.2. Nonlinear Systems

For nonlinear systems, the equi-energy volume enclosed by the system’s trajectory in phase space is not an ellipsoid. Moreover, this volume does not generally have a closed-form solution. It is possible to determine the volume either numerically or through the use of elliptic integrals [21]. However, the inability to obtain a volume in closed-form is problematic, since such a result is required for determining the generating function. For systems with weak nonlinearity, the equi-energy volume in phase space can be approximated by taking a polynomial series expansion about a parameter governing the strength of nonlinearity [7,28]. Assuming weak nonlinearity and using such an approach, a volume can be obtained in the form of a polynomial:

(22) $$V\left(E\right)=\sum_{i=1}^{N}B_{i}E^{i},$$

where $B_{i}$ are constants that are dependent upon the physical parameters of the system, such as the spring stiffnesses, modal masses, and parameters associated with the nonlinarity. Accordingly, the structure function is given by

(23) $$\Omega \left(E\right)=V^{\prime }\left(E\right)=\sum_{i=1}^{N}iBE^{i-1}=\sum_{i=1}^{N}Q_{i},$$

where $Q_{i}:=iBE^{i-1}$. The Laplace transform of Ω(E) from *E* to *s* yields the generating function:

(24) $$\Phi \left(s\right)=L\left\{\Omega \left(E\right)\right\}=L\left\{\sum_{i=1}^{N}Q_{i}\right\}=\int_{0}^{\infty }\sum_{i=1}^{N}Q_{i}dE=\sum_{i=1}^{N}\Gamma \left(i\right)Bis^{-i}.$$

From here, the procedure for determining entropy is the same as for linear systems. Khinchin’s entropy can be found using Equations (7) and (8). In general, T≠E/N for nonlinear systems.

### 3.3. Mixing Entropy

Khinchin’s entropy satisfies the entropy inequality property [7,17]:

(25) $$H\geq H^{*},$$

where *H* is the entropy of the system and $H^{*}$ is the hypothetical decoupled entropy of the system [17]. The hypothetical decoupled entropy of each subsystem, $H_{i}^{*}$, is defined as the entropy of that subsystem obtained by neglecting coupling effects. In other words, Khinchin’s definition of entropy for an isolated system is used to obtain decoupled entropies for each subsystem. The hypothetical decoupled entropy of a composite system, $H^{*}$, is given by the sum of the hypothetical decoupled entropies of its subsystems:

(26) $$H^{*}=\sum_{i=1}^{N}H_{i}^{*}.$$

The mixing entropy of a system is defined as

(27) $$H_{mix}=H-H^{*}\geq 0.$$

$H_{mix}$, mixing entropy, is the difference between the actual entropy and the hypothetical decoupled entropy of the system. Therefore, the mixing entropy is the entropy produced by the coupling of systems. When coupling is introduced between oscillators, their behavior becomes more complex. Since entropy is a measure of system disorder, the act of combining oscillators cannot cause a decrease in the total entropy. Moreover, the entropy of the system will increase if the oscillators are interacting with one another. Thus, the mixing entropy must be strictly nonnegative. This has been shown to be true for linear systems [27]. It will be demonstrated that the mixing entropy is nonnegative for the nonlinear systems considered in this paper.

## 4. Entropy of Duffing Oscillators

In this section, entropy will be examined for Duffing oscillators. In Section 4.1, a Duffing oscillator of constant energy is considered and Section 4.2 discusses a set of Duffing oscillators coupled by a linear spring.

### 4.1. A Duffing Oscillator

**Figure 1:**
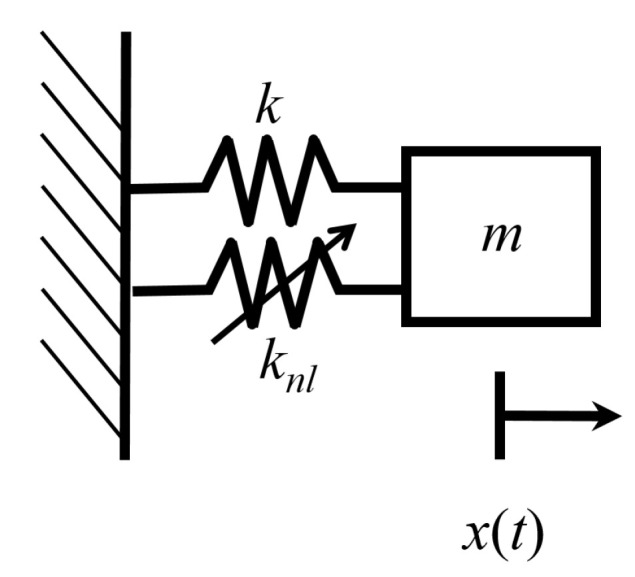
Duffing oscillator with linear stiffness *k* and nonlinear spring stiffness $k_{nl}$.

Figure 1 shows a Duffing oscillator. This oscillator has position *x*, velocity $\dot{x}$, mass *m*, spring stiffness *k*, and a nonlinear spring stiffness $k_{nl}$, associated with an anharmonic potential. The anharmonic potential, $U_{nl}\left(x\right)$, is given by

(28) $$U_{nl}\left(x\right)=\nu \frac{k_{nl}}{4}x^{4},$$

where ν is a small parameter that governs the strength of nonlinearity. The energy, *E*, of the Duffing oscillator is

(29) $$E\left(x,\dot{x}\right)=\frac{m}{2}{\dot{x}}^{2}+\frac{k}{2}x^{2}+U_{nl}\left(x\right).$$

Therefore, the equation of motion is

(30) $$m\ddot{x}+kx+\nu k_{nl}x^{3}=0.$$

Since the oscillator is not externally forced or damped, the total energy of the system is constant. The Duffing oscillator experiences a restoring force, $kx+\nu k_{nl}x^{3}$, from the linear and nonlinear springs.

For the numerical results presented in this section, the oscillator is given a linear stiffness k=1, a nonlinear stiffness $k_{nl}=1$, mass m=0.1, and an initial velocity excitation $v_{0}=\sqrt{2}$ such that the energy is given by E=0.1. All values are given in SI units.

#### 4.1.1. The Phase Volume

The equi-energy volume enclosed by the trajectory of the system cannot be determined in closed form. Therefore, a method based on series expansion and perturbation is used to calculate the volume enclosed by the equi-energy surface. Rearranging Equation (29), the velocity of the oscillator is

(31) $$\dot{x}\left(x\right)=\pm \sqrt{\frac{2}{m}}{\left(E-\frac{k}{2}x^{2}-U_{nl}\left(x\right)\right)}^{1/2}.$$

The volume enclosed by the Duffing oscillator’s trajectory in phase space is

(32) $$V\left(E\right)=4\int_{0}^{x_{M}}\dot{x}\left(x\right)dx.$$

**Figure 2:**
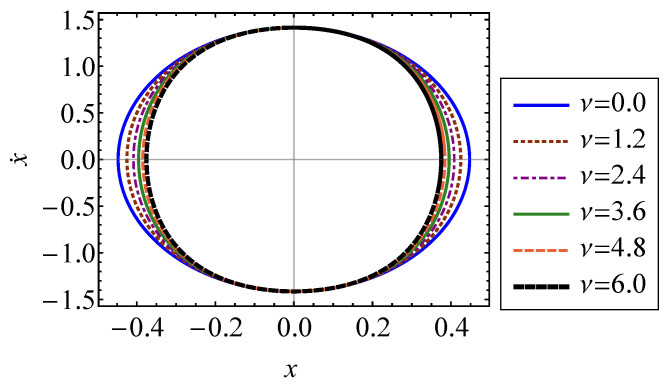
Closed-surface trajectory of a Duffing oscillator in phase space for several values of ν ranging from 0 to 6.

In Equation (32), $x_{M}$ is the abscissa of intersection between Σ and the *x* axis [15]. This is the maximum position of the oscillator. Equation (32) utilizes the symmetry of the trajectory in phase space to integrate over only the section of the surface in the first quadrant. The closed-surface trajectory, Σ, of the oscillator is shown in Figure 2 for several values of ν. It is shown that increasing the strength of nonlinearity causes the trajectory to compress symmetrically about the $\dot{x}$ axis. The system is stable since all forces acting on the oscillator are restoring forces. Therefore, increasing the strength of nonlinearity, ν, will not cause the system to deviate from its oscillatory behavior.

The integration in Equation (32) can only be carried out numerically or with the use of elliptic integrals [21]. This is problematic, because in order to determine entropy, it is necessary to have a phase-space volume, *V*, that is in closed-form with respect to the system energy, i.e., V(E). In other words, it must be possible to obtain a generating function, and thereby an entropy, from *V*. Thus, a series expansion of velocity is used to determine the phase-space volume, *V*, for weak nonlinearity, or small values of ν. In this section, a fourth order approximation is shown. One can obtain the results for lower order expansions by eliminating higher order terms from the fourth order solution. Taking a fourth order series expansion of the velocity, $\dot{x}$, about ν=0 and denoting the $i^{th}$ order term of the expansion as ${\dot{x}}^{\left[i\right]}$,

(33) $$\dot{x}\left(x\right)\approx {\dot{x}}^{\left[0\right]}+{\dot{x}}^{\left[1\right]}\nu +{\dot{x}}^{\left[2\right]}\nu^{2}+{\dot{x}}^{\left[3\right]}\nu^{3}+{\dot{x}}^{\left[4\right]}\nu^{4}=\hat{\dot{x}}\left(x\right),$$

where $\hat{\dot{x}}$ is the approximate velocity obtained via series expansion. Obtaining the coefficients, $\left\{{\dot{x}}^{\left[0\right]},{\dot{x}}^{\left[1\right]},{\dot{x}}^{\left[2\right]},{\dot{x}}^{\left[3\right]},{\dot{x}}^{\left[4\right]}\right\}$, using a Taylor series approximation yields

(34) $$\begin{array}{cc} {\dot{x}}^{\left[0\right]}=\sqrt{\frac{1}{m}}\sqrt{2E-kx^{2}},\;{\dot{x}}^{\left[1\right]}=-\frac{k_{nl}x^{4}}{4\sqrt{m(2E-kx^{2})}} & ,\;{\dot{x}}^{\left[2\right]}=-\frac{k_{nl}^{2}x^{8}}{32\sqrt{m(2E-kx^{2})}\left(2E-kx^{2}\right)},\\ \;{\dot{x}}^{\left[3\right]}=-\frac{k_{nl}^{3}x^{12}}{128\sqrt{m(2E-kx^{2})}{\left(2E-kx^{2}\right)}^{2}}, & \;{\dot{x}}^{\left[4\right]}=-\frac{5k_{nl}^{4}x^{16}}{2048\sqrt{m(2E-kx^{2})}{\left(2E-kx^{2}\right)}^{3}}. \end{array}$$

The maximum velocity, ${\dot{x}}_{M}$, is found by setting x=0 in Equation (33):

(35) $${\dot{x}}_{M}=\sqrt{\frac{2E}{m}}.$$

In order to integrate over the velocity approximated by Equation (33), the limits of integration must be determined. These are given, respectively, by zero and the maximum oscillator position, $x_{M}$. Applying a fourth order series expansion to $x_{M}$,

(36) $$x_{M}\approx x_{M}^{\left[0\right]}+x_{M}^{\left[1\right]}\nu +x_{M}^{\left[2\right]}\nu^{2}+x_{M}^{\left[3\right]}\nu^{3}+x_{M}^{\left[4\right]}\nu^{4}.$$

The perturbation method can be used to find the coefficients of the expansion of $x_{M}$ about ν=0. First, $\dot{x}$ is set to zero in Equation (29):

(37) $$E\left(x,\dot{x}\right)=\frac{k}{2}x^{2}+U_{nl}\left(x\right).$$

Then, Equation (36) is substituted into Equation (37). Retaining only the zeroth order terms with respect to ν,

(38) $$\frac{1}{2}k{\left(x_{M}^{\left[0\right]}\right)}^{2}=E.$$

Solving for $x_{M}^{\left[0\right]}$ yields

(39) $$x_{M}^{\left[0\right]}=\sqrt{\frac{2E}{k}}.$$

Then, Equation (36) is substituted into Equation (37). Retaining only up through the first order terms with respect to ν,

(40) $$\frac{1}{2}k{\left(x_{M}^{\left[0\right]}\right)}^{2}+\left(\frac{1}{4}k_{nl}{\left(x_{M}^{\left[0\right]}\right)}^{4}+kx_{M}^{\left[0\right]}x_{M}^{\left[1\right]}\right)\nu =E.$$

Substituting Equation (39) into Equation (40) and solving for $x_{M}^{\left[1\right]}$,

(41) $$x_{M}^{\left[1\right]}=-\sqrt{\frac{2E}{k}}\frac{Ek_{nl}}{2k^{2}}.$$

This procedure is applied recursively to find subsequent terms of higher order. Obtaining terms through the fourth order in ν,

(42) $$x_{M}^{\left[2\right]}=\sqrt{\frac{2E}{k}}\frac{7E^{2}k_{nl}^{2}}{8k^{4}},\;x_{M}^{\left[3\right]}=-\sqrt{\frac{2E}{k}}\frac{33E^{3}k_{nl}^{3}}{16k^{6}},\;x_{M}^{\left[4\right]}=\sqrt{\frac{2E}{k}}\frac{715E^{4}k_{nl}^{4}}{128k^{8}}.$$

Having approximated $x_{M}$, the volume enclosed by the oscillator’s trajectory in phase space is given by substituting Equation (36) into Equation (32):

(43) $$V\left(E\right)=4\int_{0}^{x_{M}^{\left[0\right]}+x_{M}^{\left[1\right]}\nu +x_{M}^{\left[2\right]}\nu^{2}+x_{M}^{\left[3\right]}\nu^{3}+x_{M}^{\left[4\right]}\nu^{4}}\hat{\dot{x}}\left(x\right)dx,$$

Assuming that ν is sufficiently small due to the weak nonlinearity and one can repesents the phase volume and a polynomial (Equation (16)), the trapezoidal rule can be used to approximate the integration in Equation (43). However, it can be verified that the higher order terms of the maximum position, $x_{M}$, have a negligible effect on the phase-space volume. See Appendix A Neglecting these terms, Equation (43) reduces to

(44) $$V\left(E\right)\approx 4\int_{0}^{x_{M}^{\left[0\right]}}\hat{\dot{x}}\left(x\right)dx.$$

Using first through fourth order expansions in velocity, the phase-space volume is obtained from Equation (44):

(45a) $$V^{\left(1\right)}\left(E\right)=\frac{E\pi }{\sqrt{km}}\left[2-\frac{3Ek_{nl}}{4k^{2}}\nu \right],$$

(45b) $$V^{\left(2\right)}\left(E\right)=\frac{E\pi }{\sqrt{km}}\left[2-\frac{3Ek_{nl}}{4k^{2}}\nu +\frac{35E^{2}k_{nl}^{2}}{32k^{4}}\nu^{2}\right],$$

(45c) $$V^{\left(3\right)}\left(E\right)=\frac{E\pi }{\sqrt{km}}\left[2-\frac{3Ek_{nl}}{4k^{2}}\nu +\frac{35E^{2}k_{nl}^{2}}{32k^{4}}\nu^{2}-\frac{1155E^{3}k_{nl}^{3}}{512k^{6}}\nu^{3}\right],$$

(45d) $$V^{\left(4\right)}\left(E\right)=\frac{E\pi }{\sqrt{km}}\left[2-\frac{3Ek_{nl}}{4k^{2}}\nu +\frac{35E^{2}k_{nl}^{2}}{32k^{4}}\nu^{2}-\frac{1155E^{3}k_{nl}^{3}}{512k^{6}}\nu^{3}+\frac{45045E^{4}k_{nl}^{4}}{8192k^{8}}\nu^{4}\right].$$

The phase-space volume of a linear oscillator with mass *m* and spring stiffness *k* is

(46) $$V_{L}=\frac{2E\pi }{\sqrt{km}}.$$

**Figure 3:**
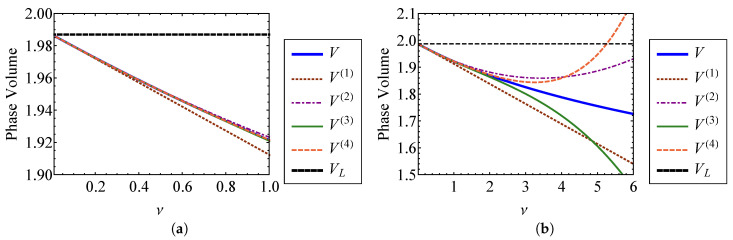
Comparison of the first through fourth order approximate volumes, $V^{\left(1\right)}$, $V^{\left(2\right)}$, $V^{\left(3\right)}$, and $V^{\left(4\right)}$, with the exact volume, *V*, for (**a**) ν=0 through ν=1 and (**b**) ν=0 through ν=6.

**Figure 4:**
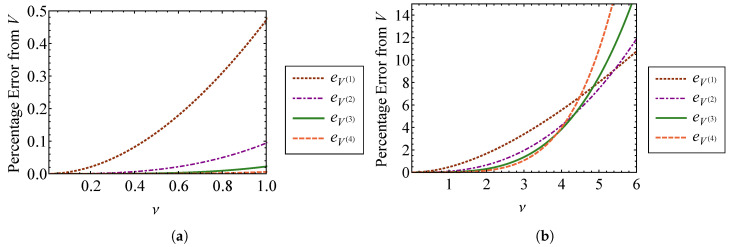
Comparison of percent error between the first through fourth order approximate volumes, $V^{\left(1\right)}$ through $V^{\left(4\right)}$, and the exact volume, *V*, for (**a**) ν=0 through ν=1 and (**b**) ν=0 through ν=6.

Comparing Equations (45) and (46), it is clear that the zeroth order terms of the nonlinear approximations are equivalent to the linear result, as expected. Figure 3 compares the volume approximations from Equation (45) to the exact volume from Equation (32) as functions of the strength of nonlinearity, ν. Figure 3a compares these volumes for ν=0 through ν=1, and Figure 3b compares them for ν=0 through ν=6. The exact volume is obtained by numerically integrating Equation (32). In each subplot, a horizontal dashed line denotes the phase-space volume of a linear oscillator, $V_{L}$, corresponding to ν=0. It is shown that each nonlinear phase volume converges with the volume of a linear oscillator as ν→0. For the small values of ν examined in Figure 3a, it can be observed that accuracy is improved by increasing the order of expansion with respect to ν. The fourth order approximation, $V^{\left(4\right)}$, is the most accurate for weak nonlinearity. However, Figure 3b shows that higher order approximations do not improve accuracy as ν becomes large. In fact, the higher order approximations deviate more from the exact volume than those of lower order for large ν. This is a consequence of expanding Equation (32) about ν=0 to find the phase volume, which assumes weak nonlinearity. Figure 4 shows the percent error between each of the phase volume approximations and the exact phase volume. The percent error in $V^{\left(i\right)}$ is defined as

(47) $$e_{V^{\left(i\right)}}=\left|100\frac{V^{\left(i\right)}-V}{V}\right|.$$

It is shown in Figure 4a that each approximation has an error of less than 0.5 percent for nonlinearity smaller than ν=1. As shown in Figure 4b, the approximation error actually increases *more* rapidly for higher order expansions as ν becomes large. The higher order expansions demonstrate improved results for small ν, as shown in Figure 4a. Sufficiently accurate results are obtained when ν=1, which is the case where the nonlinear spring stiffness is given by $k_{nl}=k$. For this case, the nonlinearity is not weak, at least in comparison to the linear spring stiffness. Therefore, this method for obtaining the phase volume can be applied to a wider range of nonlinearities than what is suggested by the relative stiffness of the nonlinear spring, if appropriate care is taken.

**Figure 5:**
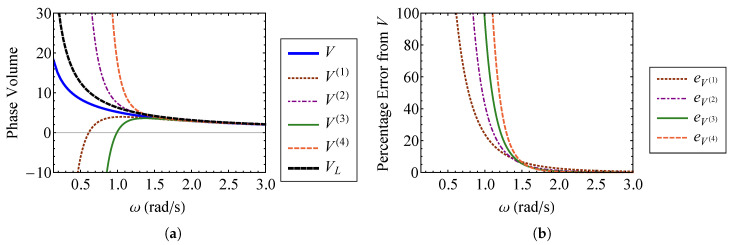
Comparison of (**a**) the first through fourth order approximate volumes, $V^{\left(1\right)}$ through $V^{\left(4\right)}$, and the exact volume, *V*, and (**b**) the error in the first through fourth order approximate volumes, for ω≈0.09 through ω=3.0.

Useful insight can be gained by fixing the strength of nonlinearity to ν=1 and varying the linear natural frequency of the system, $\omega =\sqrt{k/m}$, while maintaining $k_{nl}=k$. The phase volume approximations are compared to the exact volume in Figure 5a, and their percent errors are shown in Figure 5b. Figure 5a contains three regimes. At low frequencies, the effect of the nonlinearity ν is greater, so strong nonlinearity is achieved and the volume approximations fail. As the natural frequency increases, ν=1 becomes the weakly nonlinear case. This regime (approximately 1.5<ω<2 for ν=1), represents the region where the volume approximations become viable. Figure 5b confirms that the errors in the phase volume approximations become small at ω≈2. For ω>2, the nonlinear volume, *V*, is effectively indistinguishable from the linear system volume, $V_{L}$. These observations suggest that such an approach is especially viable for systems undergoing high frequency vibration. Such systems are the focus of SEA.

#### 4.1.2. Khinchin’s Entropy

Khinchin’s entropy will be derived using only the first and second order expansions of the phase volume about ν=0. This approach can be applied to higher order if more accuracy is desired. Defining the structure function to the first and second order with respect to ν,

(48) $$\Omega^{\left(1\right)}=V^{{\left(1\right)}^{\prime }}\left(E\right),\;\Omega^{\left(2\right)}=V^{{\left(2\right)}^{\prime }}\left(E\right),$$

the generating function is given by taking the Laplace transform of the structure function:

(49a) $$\Phi^{\left(1\right)}\left(s\right)=\frac{\pi }{\sqrt{km}}\left(\frac{2}{s}-\frac{3k_{nl}\nu }{2k^{2}s^{2}}\right),$$

(49b) $$\Phi^{\left(2\right)}\left(s\right)=\frac{\pi }{\sqrt{km}}\left(\frac{2}{s}-\frac{3k_{nl}\nu }{2k^{2}s^{2}}+\frac{105k_{nl}^{2}\nu^{2}}{16k^{4}s^{3}}\right).$$

The zeroth order term of the generating function is equivalent to the generating function of a linear oscillator [7,17]. Khinchin’s entropy must satisfy both Equation (7) and the constraint equation, Equation (8). The parameter σ is obtained to the first and second order with respect to ν by substituting Equation (49a) and Equation (49b) into Equation (8) and solving for $\sigma^{\left(1\right)}$ and $\sigma^{\left(2\right)}$, respectively. The series expansions of the temperatures, defined as T=1/σ, can be taken to the first and second order about ν=0:

(50a) $$T^{\left(1\right)}=E+\frac{3E^{2}k_{nl}\nu }{4k^{2}},$$

(50b) $$T^{\left(2\right)}=E+\frac{3E^{2}k_{nl}\nu }{4k^{2}}-\frac{39E^{3}k_{nl}^{2}\nu^{2}}{8k^{4}}.$$

The presence of weak nonlinearity results in an increase in the temperature of the Duffing oscillator relative to that of a linear oscillator, for which the temperature is given by $T_{L}=E$. Substituting Equation (50a) and Equation (50b) into Equation (7) and setting C=0 yields entropy as a function of energy:

(51a) $$\begin{array}{c} H^{\left(1\right)}=\frac{4k^{2}}{4k^{2}+3Ek_{nl}\nu }+\ln\left[\frac{2E\pi }{\sqrt{km}}\right], \end{array}$$

(51b) $$\begin{array}{c} H^{\left(2\right)}=\frac{8k^{4}}{8k^{4}+6Ek^{2}k_{nl}\nu -39E^{2}k_{nl}^{2}\nu^{2}}+\ln\left[\frac{E\pi }{\sqrt{km}}\left(2-\frac{87E^{2}k_{nl}^{2}\nu^{2}}{16k^{4}}\right)\right]. \end{array}$$

**Figure 6:**
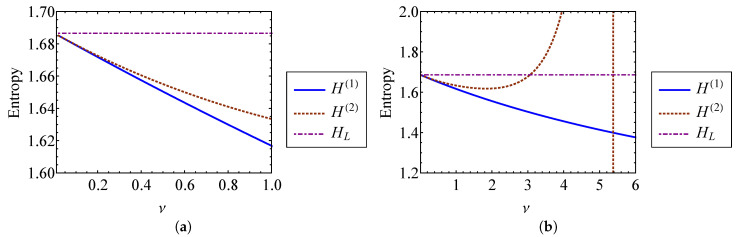
Entropies found using the first and second order phase volume approximations, $H^{\left(1\right)}$ and $H^{\left(2\right)}$, compared to the linear entropy, $H_{L}$, for (**a**) ν=0 through ν=1 and (**b**) ν=0 through ν=6.

Higher order terms are neglected in Equation (51). The entropies from Equation (51) are compared to the entropy of a linear oscillator [7,17] (ν=0) in Figure 6. Figure 6a shows that $H^{\left(1\right)}$ and $H^{\left(2\right)}$ are indistinguishable from one another for ν<0.2. This is expected, since both results should be accurate for a weakly nonlinear oscillator. Figure 6b shows that $H^{\left(1\right)}$ and $H^{\left(2\right)}$ diverge rapidly as the strength of nonlinearity grows beyond ν≈1. For ν>1, the assumption of weak nonlinearity fails, and this approach becomes unreliable.

### 4.2. Coupled Duffing Oscillators

**Figure 7:**
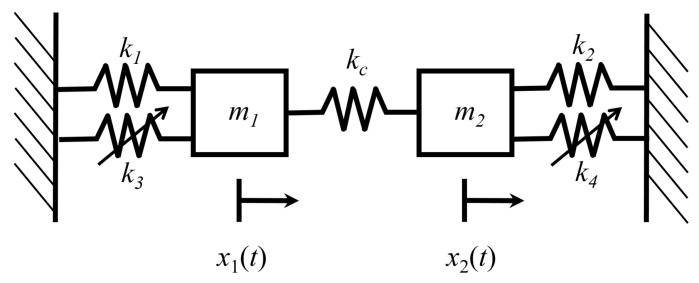
Duffing oscillators coupled by a linear spring of stiffness $k_{c}$.

Figure 7 shows a two-degree-of-freedom system of coupled Duffing oscillators [15,21]. Each oscillator has a spring with a linear restoring force as well as a nonlinear spring with a cubic restoring force. The oscillators are coupled by a linear spring with stiffness $k_{c}$. The total energy of the system is

(52) $$\begin{array}{cc} E & =\frac{m_{1}}{2}{\dot{x}}_{1}^{2}+\frac{1}{2}\left(k_{1}+k_{c}\right)x_{1}^{2}+\nu \frac{k_{3}}{4}x_{1}^{4}+\frac{m_{2}}{2}{\dot{x}}_{2}^{2}+\frac{1}{2}\left(k_{2}+k_{c}\right)x_{2}^{2}+\nu \frac{k_{4}}{4}x_{2}^{4}-k_{c}x_{1}x_{2}\\ & =E_{1}+E_{2}+E_{12}, \end{array}$$

where the oscillator energies are given by

(53) $$E_{1}\left(x_{1},{\dot{x}}_{1}\right)=\frac{m_{1}}{2}{\dot{x}}_{1}^{2}+\frac{1}{2}\left(k_{1}+k_{c}\right)x_{1}^{2}+\nu \frac{k_{3}}{4}x_{1}^{4},\;E_{2}\left(x_{2},{\dot{x}}_{2}\right)=\frac{m_{2}}{2}{\dot{x}}_{2}^{2}+\frac{1}{2}\left(k_{2}+k_{c}\right)x_{2}^{2}+\nu \frac{k_{4}}{4}x_{2}^{4},$$

and the energy associated with the coupling of the oscillators is

(54) $$E_{12}=-k_{c}x_{1}x_{2}.$$

In Equations (52) through (53), $k_{1}$ and $k_{2}$ are the linear spring stiffnesses of oscillators 1 and 2, respectively. $k_{3}$ and $k_{4}$ are the nonlinear spring stiffness of oscillators, $m_{1}$ and $m_{2}$ are the oscillator masses, and ν remains a dimensionless scaling parameter that governs the strength of nonlinearity of the oscillators.

In this analysis, it is assumed that the oscillators are weakly coupled, i.e., the energy associated with coupling is small compared to the subsystem energies. That is to say,

(55) $$E=E_{1}+E_{2}+E_{12}\approx E_{1}+E_{2}.$$

For the numerical results presented in this section, the following values are selected: linear spring stiffnesses $k_{1}=k_{2}=1$, nonlinear spring stiffnesses $k_{3}=k_{4}=1$, oscillator masses $m_{1}=m_{2}=0.1$, and coupling spring stiffness $k_{c}=0.01$. All values are given in SI units. Values of ν will be selected such that the system satisfies the assumption of weak nonlinearity. Similarly, the coupling stiffness $k_{c}$ is sufficiently small for the system to satisfy the weak coupling assumption, but not so small that the oscillators do not interact with one another.

**Figure 8:**
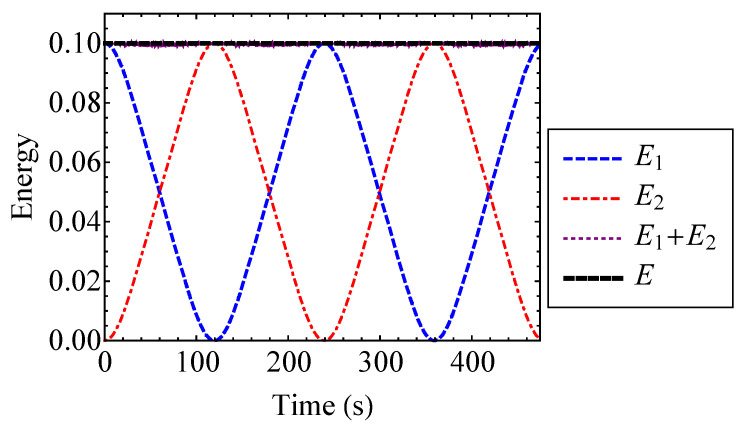
Energies $E_{1}$ and $E_{2}$ of the Duffing oscillators compared to their sum, $E_{1}+E_{2}$, and the total energy, *E*, for ν=0.2.

The weak coupling assumption, Equation (55), enables the use of the law of composition of the generating function from Equation (6). The energies of the oscillators are compared to the total system energy for ν=0.2 in Figure 8. Figure 8 verifies that the total energy of the system is approximately equal to the sum of the oscillator energies. Thus, the weak coupling assumption is valid for the selected strength of nonlinearity.

Using Lagrangian mechanics, the equations of motion for the coupled Duffing system are

(56a) $$\left(k_{1}+k_{c}\right)x_{1}+\nu k_{3}x_{1}^{3}+m_{1}{\ddot{x}}_{1}-k_{c}x_{2}=0,$$

(56b) $$\left(k_{2}+k_{c}\right)x_{2}+\nu k_{4}x_{2}^{3}+m_{2}{\ddot{x}}_{2}-k_{c}x_{1}=0.$$

#### 4.2.1. Entropy of the Coupled Duffing System

The entropy of the coupled Duffing system will be found by utilizing the law of composition from Equation (6) to determine the generating function of the system. The generating function of the entire system, Φ, is taken to be the product of the generating functions of each oscillator, $\Phi_{1}$ and $\Phi_{2}$. The generating functions of each oscillator are found to the first and second order by substituting the appropriate physical parameters into Equation (49). Then, the generating function of the entire system is found by taking the product of the first and second order generating functions of each oscillator:

(57a) $$\Phi^{\left(1\right)}\left(s\right)=\Phi_{1}^{\left(1\right)}\left(s\right)\Phi_{2}^{\left(1\right)}\left(s\right),$$

(57b) $$\Phi^{\left(2\right)}\left(s\right)=\Phi_{1}^{\left(2\right)}\left(s\right)\Phi_{2}^{\left(2\right)}\left(s\right).$$

Substituting Equation (49) for each oscillator into Equation (57) and and simplifying it results in

(58a) $$\Phi^{\left(1\right)}\left(s\right)=\frac{\pi^{2}}{\sqrt{k_{1}k_{2}m_{1}m_{2}}}\left(\frac{4}{s^{2}}-\frac{3(k_{2}^{2}k_{3}+k_{1}^{2}k_{4})}{k_{1}^{2}k_{2}^{2}s^{3}}\nu \right),$$

(58b) $$\begin{array}{c} \Phi^{\left(2\right)}\left(s\right)=\frac{\pi^{2}}{\sqrt{k_{1}k_{2}m_{1}m_{2}}}\left(\frac{4}{s^{2}}-\frac{3(k_{2}^{2}k_{3}+k_{1}^{2}k_{4})}{k_{1}^{2}k_{2}^{2}s^{3}}\nu +\frac{105\left(k_{2}^{4}k_{3}^{2}+k_{1}^{4}k_{4}^{2}\right)+18k_{1}^{2}k_{2}^{2}k_{3}k_{4}}{8k_{1}^{4}k_{2}^{4}s^{4}}\nu^{2}\right). \end{array}$$

The temperature $T^{\left(1\right)}$ can be found by substituting the generating function from Equation (57a) into the constraint equation, Equation (8), to find $\sigma^{\left(1\right)}$. Then, $T^{\left(1\right)}=1/\sigma^{\left(1\right)}$. $T^{\left(2\right)}$ can be found similarly by solving for $\sigma^{\left(2\right)}$ using Equation (57b). When finding $\sigma^{\left(2\right)}$, the series expansion of the left side of Equation (8) is taken to the second order with respect to ν. The resulting equation is then solved for $\sigma^{\left(2\right)}$. Taking the series expansions of $T^{\left(1\right)}$ and $T^{\left(2\right)}$ to the first and second order about ν=0 yields

(59a) $$T^{\left(1\right)}\approx \frac{E}{2}+\frac{3E^{2}\left(k_{2}^{2}k_{3}+k_{1}^{2}k_{4}\right)}{32k_{1}^{2}k_{2}^{2}}\nu ,$$

(59b) $$T^{\left(2\right)}\approx \frac{E}{2}+\frac{3E^{2}\left(k_{2}^{2}k_{3}+k_{1}^{2}k_{4}\right)}{32k_{1}^{2}k_{2}^{2}}\nu -\frac{3E^{3}\left(29k_{2}^{4}k_{3}^{2}-6k_{1}^{2}k_{2}^{2}k_{3}k_{4}+29k_{1}^{4}k_{4}^{2}\right)}{256k_{1}^{4}k_{2}^{4}}\nu^{2}.$$

The temperatures in Equation (59) agree in their first and zeroth order terms, such that $T^{\left(2\right)}=T^{\left(1\right)}+T^{\left[2\right]}\left(\nu \right)$, where $T^{\left[2\right]}\left(\nu \right)$ is the second order term with respect to ν. The zeroth order term is identical to the result for linear oscillators, which is consistent with previous results by Carcaterra [7]. The same approach that is used here to obtain the temperature can be applied to systems with many degrees of freedom. This would allow for the extension of SEA into the nonlinear regime, which is a subject of priority for future work.

Using Equation (7) and setting C=0, the entropy of the coupled Duffing system is found by dropping all but the the first and second order terms, respectively, from the following equations:

(60a) $$H^{\left(1\right)}\left(E\right)=\ln\left[e^{E/T^{\left(1\right)}}\Phi^{\left(1\right)}\left(1/T^{\left(1\right)}\right)\right],$$

(60b) $$H^{\left(2\right)}\left(E\right)=\ln\left[e^{E/T^{\left(2\right)}}\Phi^{\left(2\right)}\left(1/T^{\left(2\right)}\right)\right].$$

This yields

(61a) $$H^{\left(1\right)}\left(E\right)=\frac{32k_{1}^{2}k_{2}^{2}}{16k_{1}^{2}k_{2}^{2}+3E\left(k_{2}^{2}k_{3}+k_{1}^{2}k_{4}\right)\nu }+\ln\left[\frac{E\pi^{2}}{\sqrt{k_{1}k_{2}m_{1}m_{2}}}\right],$$

(61b) $$H^{\left(2\right)}\left(E\right)=D^{\left(2\right)}+\ln\left[\frac{E\pi^{2}}{\sqrt{k_{1}k_{2}m_{1}m_{2}}}\left(1-\frac{3E^{2}\left(61k_{2}^{4}k_{3}^{2}+61k_{1}^{4}k_{4}^{2}-6k_{1}^{2}k_{2}^{2}k_{3}k_{4}\right)}{256k_{1}^{4}k_{2}^{4}}\nu^{2}\right)\right],$$

where $D^{\left(2\right)}$ is the second order series expansion of $E/T^{\left(2\right)}$ about ν:

(62) $$D^{\left(2\right)}=2-\frac{3\left(k_{2}^{2}k_{3}+k_{1}^{2}k_{4}\right)}{8k_{1}^{2}k_{2}^{2}}\nu +\frac{3E^{2}\left(61k_{2}^{4}k_{3}^{2}+61k_{1}^{4}k_{4}^{2}-6k_{1}^{2}k_{2}^{2}k_{3}k_{4}\right)}{128k_{1}^{4}k_{2}^{4}}\nu^{2}.$$

While Equation (62) does not have to be obtained by series expansion, this produces a mathematically elegant result without a significant loss of accuracy.

#### 4.2.2. Mixing Entropy of the Coupled Duffing System

The entropy inequality property, Equation (27), states the mixing entropy of a composite system must be strictly nonnegative, where the mixing entropy is defined as the difference between the actual entropy of the system and the sum of the hypothetical decoupled entropies of the subsystems. The hypothetical decoupled entropy of each oscillator is the entropy that is found by neglecting the entropy that is generated by coupling. In other words, Equation (51) yields the hypothetical decoupled entropy of each oscillator with the substitution of the appropriate physical parameters. The hypothetical decoupled entropies of the oscillators are denoted as $H_{1}^{*(1)}\left(E_{1}\right)$ and $H_{2}^{*(1)}\left(E_{2}\right)$ using the first order approximation with respect to ν, and as $H_{1}^{*(2)}\left(E_{1}\right)$ and $H_{2}^{*(2)}\left(E_{2}\right)$ using the second order approximation. The total hypothetical decoupled entropy is denoted as

(63) $$H^{*(1)}\left(E_{1},E_{2}\right)=H_{1}^{*(1)}\left(E_{1}\right)+H_{2}^{*(1)}\left(E_{2}\right),\;H^{*(2)}\left(E_{1},E_{2}\right)=H_{1}^{*(2)}\left(E_{1}\right)+H_{2}^{*(2)}\left(E_{2}\right),$$

using the first and second order approximations with respect to ν, respectively. From here, the mixing entropy is the difference between the actual entropy of the system and the total hypothetical decoupled entropy:

(64) $$H_{mix}^{\left(1\right)}=H^{\left(1\right)}\left(E\right)-H^{*(1)}\left(E_{1},E_{2}\right),\;H_{mix}^{\left(2\right)}=H^{\left(2\right)}\left(E\right)-H^{*(2)}\left(E_{1},E_{2}\right).$$

It is important to note that the actual entropy, *H*, is a function of the total energy of the system. Since the system is conservative, the total energy and entropy are both constant. However, the hypothetical decoupled entropy is a function of both subsystem energies, and is therefore a function of time. Consequently, the mixing entropy is a function of time as well.

**Figure 9:**
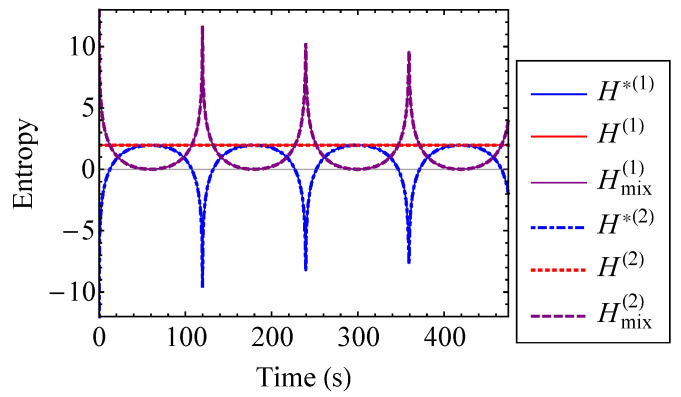
Actual entropy, *H*, total hypothetical decoupled entropy, $H^{*}$, and mixing entropy, $H_{mix}$, of the coupled Duffing system approximated to the first and second order for ν=0.2.

The actual entropy, total hypothetical decoupled entropy, and mixing entropy of the coupled Duffing system are plotted to the first and second order for ν=0.2 in Figure 9. For ν=0.2, the first and second order approximations are indistinguishable for the actual, hypothetical decoupled, and mixing entropies. Thus, a first order approximation with respect to ν is sufficient in this case. Figure 9 verifies that the actual entropy of the system is constant, while the hypothetical decoupled entropy is time-dependent. As stated by the entropy inequality property, the mixing entropy is nonnegative. The mixing entropies shown in Figure 9 are similar to the result that one would see for a linear oscillator [17]. When the oscillator energies are equivalent, the coupled Duffing system is indistinguishable from a pair of uncoupled Duffing oscillators. Therefore, the relative minima of the mixing entropy correspond to the points at which $E_{1}=E_{2}$. This is because there is no exchange of energy at the instances when the oscillator energies are equal to one another. In other words, there is no coupling contribution to the total entropy at these instances [17]. Thus, the mixing entropy is minimized. Because of its relationship with energy, the mixing entropy is a useful tool for understanding the qualitative behavior of complex systems with many degrees of freedom.

## 5. Entropy of Henon–Heiles Oscillators

In this section, Khinchin’s entropy will be found for weakly nonlinear Henon–Heiles oscillators [21,24]. Section 5.1 discusses an oscillator with a third order anharmonic potential. This oscillator is one of the nonlinear components of the Henon–Heiles system. While the anharmonic potential of a Duffing oscillator is a restoring force, the nonlinear potential considered in Section 5.1 is repulsive. Section 5.2 discusses a set of Henon–Heiles oscillators.

### 5.1. Single Degree of Freedom Oscillator with Third Order Anharmonic Potential

Consider an oscillator with position *x*, velocity $\dot{x}$, mass *m*, spring stiffness *k*, and a nonlinear spring stiffness $k_{nl}$ associated with a third order anharmonic potential. The anharmonic potential, $U_{nl}\left(x\right)$, is given by

(65) $$U_{nl}\left(x\right)=-\nu k_{nl}\frac{x^{3}}{3},$$

where ν is a small parameter that governs the strength of nonlinearity, and $k_{nl}$ is the nonlinear spring stiffness. When this oscillator is nonlinearly coupled to a simple oscillator, the configuration forms a set of Henon–Heiles oscillators [21]. The energy, *E*, of the nonlinear oscillator is

(66) $$E\left(x,\dot{x}\right)=\frac{m}{2}{\dot{x}}^{2}+\frac{k}{2}x^{2}+U_{nl}\left(x\right),$$

such that the equation of motion is

(67) $$m\ddot{x}+kx-\nu k_{nl}x^{2}=0.$$

The negative quadratic term in Equation (67) is a repulsive force. Consequently, one of the poles of this system is nonnegative. This introduces the possibility of the system becoming unstable, which is not present for a Duffing oscillator described by Equation (30). The potential for system instability results in certain limitations, which are discussed in Section 5.1.1.

The following values are selected for the numerical results presented in Section 5.1: linear spring stiffness k=1, oscillator mass m=1, initial velocity $v_{0}=\sqrt{2}$, and nonlinear spring stiffness $k_{nl}=1$, with varying strength of nonlinearity, ν. All values are given in SI units.

#### 5.1.1. The Phase Volume

Solving Equation (66) for $\dot{x}$, the velocity of the nonlinear oscillator described by Equation (67) is

(68) $$\dot{x}\left(x\right)=\pm \sqrt{\frac{2}{m}}{\left(E-\frac{k}{2}x^{2}+\nu k_{nl}\frac{x^{3}}{3}\right)}^{1/2}.$$

**Figure 10:**
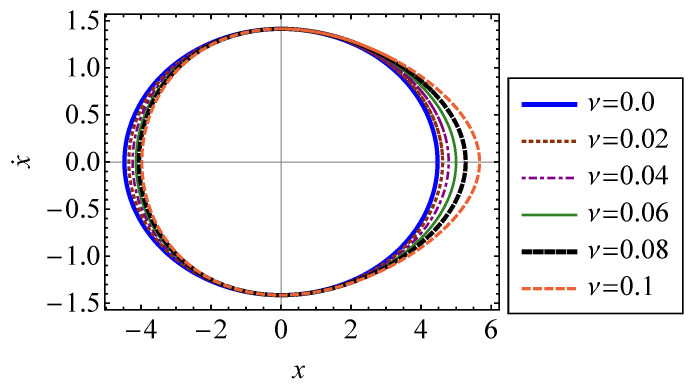
Closed-surface trajectory in phase space of an oscillator with velocity given by Equation (68). Trajectories are shown for several values of ν ranging from 0 to 0.1. All values of ν are less than the critical value, $\nu_{c}$.

**Figure 11:**
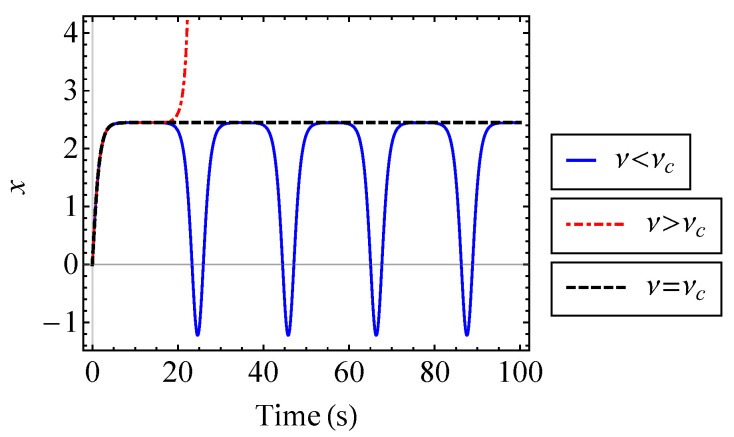
Position of the oscillator described by Equation (67), shown for ν just above, below, and equal to the critical value, $\nu_{c}$.

The phase-space trajectory of the oscillator is shown in Figure 10 using Equation (68) for several values of ν. The third order anharmonic potential, Equation (65), introduces asymmetry into the phase-space trajectory of the oscillator. It is shown that with increasing nonlinearity, the trajectory of the system expands asymmetrically along the *x*-axis. Moreover, the instability of the system causes the solution to blow up when ν is greater than a certain value, $\nu_{c}$. For $\nu >\nu_{c}$, the initial velocity imparts sufficient energy into the system for the oscillator to escape from the origin [21]. When this occurs, the trajectory no longer forms a closed surface in phase space. When $\nu <\nu_{c}$, the behavior is oscillatory, and at $\nu =\nu_{c}$, the oscillator settles into an unstable equilibrium position. The position of the oscillator is shown over time for $\nu <\nu_{c}$, $\nu =\nu_{c}$, and $\nu >\nu_{c}$ in Figure 11 [21]. To find the critical value, $\nu_{c}$, it is necessary to find the unstable equilibrium point. The equilibrium points of the system are where the position of the oscillator is constant, i.e., $\ddot{x}=\dot{x}=0$ [29]. That is, Equation (67) becomes

(69) $$kx-\nu k_{nl}x^{2}=0,$$

which is satisfied when

(70) $$x=0,\;x=x_{c}=\frac{k}{k_{nl}\nu }.$$

The point $\left\{x=0,\dot{x}=0\right\}$ is a stable equilibrium point, while $\left\{x=x_{c},\dot{x}=0\right\}$ is an unstable equilibrium point. The critical value of the strength of nonlinearity, $\nu_{c}$, can be found by substituting the position and velocity at unstable equilibrium, $\left\{x_{c},0\right\}$, into Equation (66) and solving for ν. This yields

(71) $$\nu_{c}=\sqrt{\frac{k}{6E}}\frac{k}{k_{nl}}.$$

**Figure 12:**
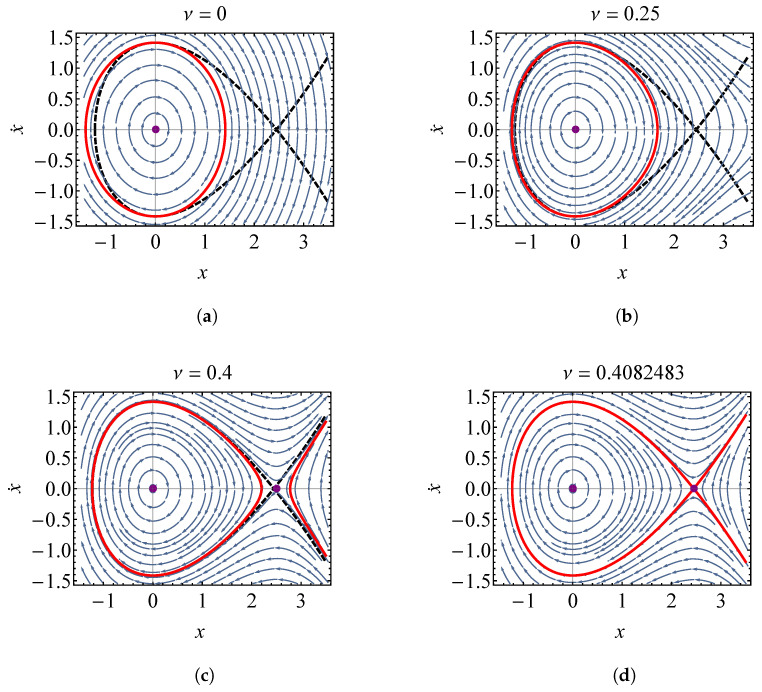
Phase portrait of the oscillator with quadratic repulsive force $\nu k_{nl}x^{2}$ described by Equation (67) for (**a**) ν=0 (**b**) ν=0.25 (**c**) ν=0.4 and (**d**) $\nu =\nu_{c}=1/\sqrt{6}$. The dashed line represents the separatrix when $\nu =\nu_{c}$, the solid line is the system trajectory when E=1, and the arrows represent trajectories at other energies. Equilibrium points are shown as dots.

For the physical parameters and initial conditions selected in this section, $\nu_{c}=1/\sqrt{6}\approx 0.40825$. To provide some insight into how the behavior of the oscillator changes with increasing nonlinearity, phase portraits for ν=0, ν=0.25, ν=0.4, and $\nu =1/\sqrt{6}$ are shown in Figure 12. The point markers on the plot denote equilibrium points, the dashed line denotes the separatrix when $\nu =\nu_{c}$, and the solid line is the system trajectory when E=1. When ν=0, the system is stable and linear, and the only equilibrium point is at the origin. The streamlines within the solid line represent trajectories with E<1, and those outside of the solid line represent trajectories with E>1. As ν is increased, a saddle point develops at $p_{2}=\left\{x_{c},0\right\}$. Since $x_{c}=k/k_{nl}\nu$, the saddle point approaches the origin as the strength of nonlinearity increases. Figure 12d shows that when $\nu =\nu_{c}=1/\sqrt{6}$, the trajectory of the oscillator is along the separatrix, and the oscillator reaches unstable equilibrium. It is shown that trajectories within the left loop of the E=1 trajectory (trajectories with E<1) are stable, whereas those with E≥1 are unstable.

As a result of the instability of the oscillator, ν must be less than $\nu_{c}$ for the trajectory to form a closed surface. In the case of weak nonlinearity, this is generally true, and a phase-space volume can be found. The trajectory is asymmetric, and thus the magnitude of the most positive position, $x_{M}^{+}$, is not the same as the magnitude of the most negative position, $x_{M}^{-}$. Therefore, the equi-energy volume enclosed by the phase-space trajectory is

(72) $$V\left(E\right)=2\int_{x_{M}^{-}}^{x_{M}^{+}}\dot{x}\left(x\right)dx.$$

In this section, an approximation of the phase volume is obtained to the fourth order with respect to ν. Taking the series expansion of the integrand of Equation (72) yields

(73) $$\dot{x}\left(x\right)\approx {\dot{x}}^{\left[0\right]}+{\dot{x}}^{\left[1\right]}\nu +{\dot{x}}^{\left[2\right]}\nu^{2}+{\dot{x}}^{\left[3\right]}\nu^{3}+{\dot{x}}^{\left[4\right]}\nu^{4}=\hat{\dot{x}}\left(x\right),$$

where

(74) $$\begin{array}{cc} {\dot{x}}^{\left[0\right]}=\sqrt{\frac{2E-kx^{2}}{m}},\;{\dot{x}}^{\left[1\right]}=\frac{k_{nl}x^{3}}{3\sqrt{m(2E-kx^{2})}}, & \;{\dot{x}}^{\left[2\right]}=-\frac{k_{nl}^{2}x^{6}}{18\sqrt{m(2E-kx^{2})}\left(2E-kx^{2}\right)},\\ {\dot{x}}^{\left[3\right]}=\frac{k_{nl}^{3}x^{9}}{54\sqrt{m(2E-kx^{2})}{\left(2E-kx^{2}\right)}^{2}}, & \;{\dot{x}}^{\left[4\right]}=-\frac{5k_{nl}^{4}x^{12}}{648\sqrt{m(2E-kx^{2})}{\left(2E-kx^{2}\right)}^{3}}. \end{array}$$

A perturbation approach is used to approximate $x_{M}^{+}$ and $x_{M}^{-}$ to fourth order. Taking Equation (66) and setting $\dot{x}=0$, the perturbation method yields two possible solutions, $x_{M}^{+}$ and $x_{M}^{-}$:

(75) $$x_{M}^{+}\approx \pm \left(\sqrt{\frac{2E}{k}}+\frac{2E^{}k_{nl}}{3k^{2}}\nu +\sqrt{\frac{2E}{k}}\frac{5Ek_{nl}^{2}}{9k^{3}}\nu^{2}+\frac{32E^{2}k_{nl}^{3}}{27k^{5}}\nu^{3}+\sqrt{\frac{2E}{k}}\frac{77E^{2}k_{nl}^{4}}{54k^{6}}\nu^{4}\right).$$

Equation (72) cannot be integrated directly by substituting Equation (75) and the exact velocity, $\dot{x}$. Consequently, the phase volume is obtained by integrating the approximate velocity, $\hat{\dot{x}}$, over the approximated limits of integration, $x_{M}^{+[0]}$ and $x_{M}^{-[0]}$:

(76) $$V\left(E\right)=2\int_{x_{M}^{-[0]}}^{x_{M}^{+[0]}}\hat{\dot{x}}\left(x\right)dx.$$

The phase-space volume of the nonlinear oscillator described by Equation (67) is given from the first through fourth order with respect to ν by

(77a) $$V^{\left(1\right)}\left(E\right)=\frac{2E\pi }{\sqrt{km}},$$

(77b) $$V^{\left(2\right)}\left(E\right)=\frac{E\pi }{\sqrt{km}}\left(2+\frac{5Ek_{nl}^{2}}{6k^{3}}\nu^{2}\right),\;V^{\left(3\right)}\left(E\right)=V^{\left(2\right)}\left(E\right),$$

(77c) $$V^{\left(4\right)}\left(E\right)=\frac{E\pi }{\sqrt{km}}\left(2+\frac{5Ek_{nl}^{2}}{6k^{3}}\nu^{2}+\frac{385E^{3}k_{nl}^{4}}{216k^{6}}\nu^{4}\right).$$

**Figure 13:**
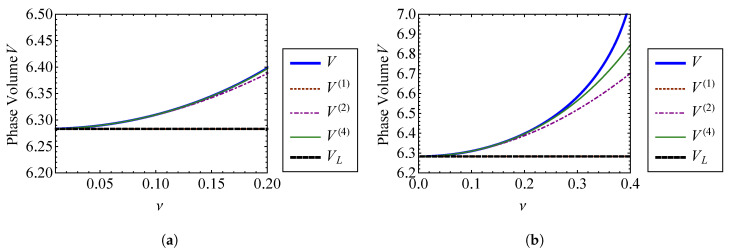
Comparison of $V^{\left(1\right)},V^{\left(2\right)},$ and $V^{\left(4\right)}$ for the oscillator described by Equation (67) with the exact volume, *V*, for (**a**) ν=0 through ν=0.1 and (**b**) ν=0 through ν=0.4.

**Figure 14:**
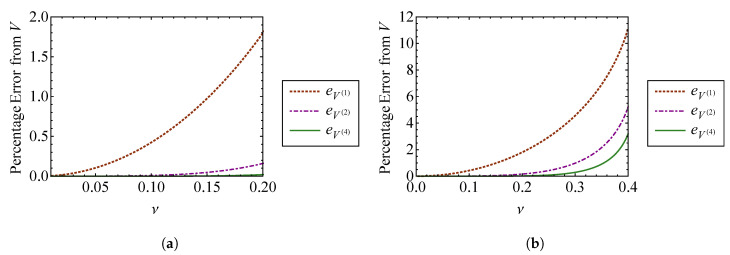
Comparison of the percent errors from the exact volume, *V*, in $V^{\left(1\right)},\;V^{\left(2\right)},$ and $V^{\left(4\right)}$ for the oscillator described by Equation (67) for (**a**) ν=0 through ν=0.2 and (**b**) ν=0 through ν=0.4.

$V^{\left(1\right)}$ is equivalent to the linear oscillator volume, $V_{L}$, from Equation (46). Thus, it is necessary to approximate the phase-space volume to the second order with respect to ν to see an improvement over the linear result. Additionally, the third order approximation, $V^{\left(3\right)}$, is identical to the second order result. The volume approximations and their percent errors are compared in Figure 13 and Figure 14, respectively. It is shown that the phase volume of this oscillator increases with ν. Figure 13 verifies that the first order approximation, $V^{\left(1\right)}$, is the same as the linear result. The second and fourth order volume approximations, $V^{\left(2\right)}$ and $V^{\left(4\right)}$, are indistinguishable for small ν. Unlike the result for the Duffing oscillator, $V^{\left(4\right)}$ has improved accuracy over $V^{\left(2\right)}$ for larger values of ν, as shown in Figure 14. This does not hold true in general. For weakly nonlinear systems, the difference in accuracy between the two approximations is negligible.

**Figure 15:**
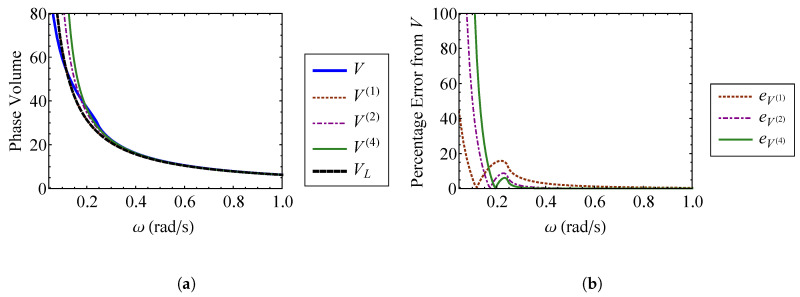
Comparison of (**a**) the first through fourth order approximate volumes, $V^{\left(1\right)}$ through $V^{\left(4\right)}$, and the exact volume, *V*, and (**b**) the error in the first through fourth order approximate volumes for ω≈0.04 through ω=1.0

As for the Duffing oscillator, insight can be gained by fixing the strength of nonlinearity to a set value while varying the linear natural frequency, $\omega =\sqrt{k/m}$, of the system. ν=0.1, and enforcing $k_{nl}=k$. The phase volume approximations are compared to the exact volume in Figure 15a, while their percent errors are shown in Figure 15b. Figure 15a contains three regimes. At low frequencies, the effect of the nonlinearity ν is large enough that the the volume approximations fail. The nonlinearity becomes weaker as the natural frequency increases. Figure 15b shows that the errors in the phase volume approximations become small at ω≈0.4. For ω>0.4, the nonlinear volume, *V*, is indistinguishable from the linear system volume, $V_{L}$.

#### 5.1.2. Khinchin’s Entropy

Since the error in both $V^{\left(2\right)}$ and $V^{\left(4\right)}$ is less than one percent for small ν, entropy can be derived with sufficient accuracy using only the second order phase-volume. Despite this, the second and fourth order approximations are compared to one another in this section for demonstrative purposes. The same approach can be applied using higher order approximations if more accuracy is desired. Defining the structure function as

(78) $$\Omega^{\left(2\right)}=V^{{\left(2\right)}^{\prime }}\left(E\right),\;\Omega^{\left(4\right)}=V^{{\left(4\right)}^{\prime }}\left(E\right),$$

the generating function can be obtained to the second and fourth order with respect to ν by taking the Laplace transform of Equation (78):

(79a) $$\Phi^{\left(2\right)}\left(s\right)=\frac{\pi }{\sqrt{km}}\left(\frac{2}{s}+\frac{5k_{nl}^{2}}{3k^{3}s^{2}}\nu^{2}\right),$$

(79b) $$\Phi^{\left(4\right)}\left(s\right)=\frac{\pi }{\sqrt{km}}\left(\frac{2}{s}+\frac{5k_{nl}^{2}}{3k^{3}s^{2}}\nu^{2}+\frac{385k_{nl}^{4}}{36k^{6}s^{3}}\nu^{4}\right).$$

Substituting Equations (79a) and (79b) into Equation (8) to find σ, the temperature is obtained using T=1/σ. Taking the series expansion of the temperature to the second and fourth order about ν=0,

(80a) $$T^{\left(2\right)}=E-\frac{5E^{2}k_{nl}^{2}\nu^{2}}{6k^{3}},$$

(80b) $$T^{\left(4\right)}=E-\frac{5E^{2}k_{nl}^{2}\nu^{2}}{6k^{3}}-\frac{155E^{3}k_{nl}^{4}\nu^{4}}{18k^{6}}.$$

While the presence of nonlinearity causes temperature to increase for the Duffing oscillator, it results in a decrease in temperature for an oscillator with a repulsive quadratic force. Setting C=0, the temperatures from Equation (80) can be substituted into Equation (7) to calculate entropy of the nonlinear oscillator, $H^{\left(2\right)}$ and $H^{\left(4\right)}$, as

(81a) $$\begin{array}{c} H^{\left(2\right)}=\frac{6k^{3}}{6k^{3}-5Ek_{nl}^{2}\nu^{2}}+\ln\left[\frac{2E\pi }{\sqrt{km}}\right], \end{array}$$

(81b) $$\begin{array}{c} H^{\left(4\right)}=\frac{18k^{6}}{18k^{6}-15Ek^{3}k_{nl}^{2}\nu^{2}-155E^{2}k_{nl}^{4}\nu^{4}}+\ln\left[\frac{E\pi }{\sqrt{km}}\left(2-\frac{335E^{2}k_{nl}^{4}}{36k^{6}}\nu^{4}\right)\right]. \end{array}$$

**Figure 16:**
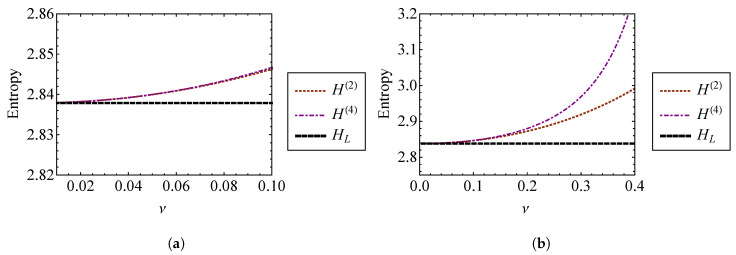
Entropies $H^{\left(2\right)}$ and $H^{\left(4\right)}$ found using second and fourth order phase volume approximations for (**a**) ν=0 through ν=0.1 and (**b**) ν=0 through ν=0.4.

Since the energy of the system is constant, the total entropy of the system is constant over time. Equation (81) gives the entropy of an oscillator with a repulsive quadratic force, $\nu k_{nl}x^{2}$, using the second and fourth order approximations of the temperature with respect to ν. These entropies are compared to the entropy of a linear oscillator in Figure 16. Figure 16a shows that for small ν, the approximations are nearly indistinguishable. As ν becomes large, the solutions diverge from one another. When ν=0.4, $H^{\left(2\right)}$ is approximately 4.2% larger than the linear result, while $H^{\left(4\right)}$ is 8.6% larger. It is expected that the fourth order approximation, $H^{\left(4\right)}$, is the more accurate of the two, since the phase volume used for this approximation has a smaller percent error than the second order solution (Figure 14b). Entropy can be thought of as a measure of chaos or disorder. Under this frame of thought, the entropy increase associated with an increase in the strength of nonlinearity, ν, can be interpreted as a result of the greater system complexity.

### 5.2. Henon–Heiles Oscillators

**Figure 17:**
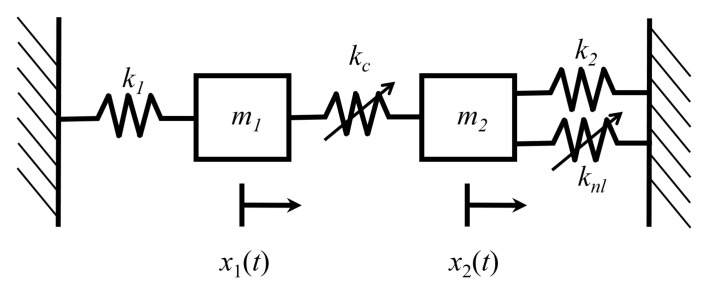
Set of Henon–Heiles oscillators.

Figure 17 shows a set of Henon–Heiles oscillators [21,24]. The Henon–Heiles system consists of two oscillators. The first is a simple linear oscillator, and the second is an oscillator with the nonlinear potential defined in Equation (65). The oscillator masses are $m_{1}$ and $m_{2}$, respectively. The spring stiffness of the first oscillator is $k_{1}$, while the second oscillator has a linear spring stiffness, $k_{2}$, and nonlinear spring stiffness $k_{nl}$. The oscillators are coupled by a nonlinear spring with stiffness $k_{c}$.

To obtain the numerical results of this section, the oscillators are given the following properties: spring stiffnesses $k_{1}=k_{2}=k_{nl}=0.1$, oscillator masses $m_{1}=m_{2}=1$, coupling spring stiffness $k_{c}=0.01k_{1}$, strength of nonlinearity ν=0.1, and initial velocities $v_{10}=1/3,v_{20}=1/\left(3\sqrt{2}\right)$ of the first and second oscillators. These initial velocities are such that the total energy is E=1/12. All values are given in SI units. This choice of physical parameters ensures that there is sufficient interaction between the oscillators for this analysis to be meaningful, while also satisfying the assumption of weak coupling and preserving the stability of the system.

The energy of the Henon–Heiles system is

(82) $$E=\frac{m_{1}}{2}{\dot{x_{1}}}^{2}+\frac{k_{1}}{2}x_{1}^{2}+\frac{m_{2}}{2}{\dot{x_{2}}}^{2}+\frac{k_{2}}{2}x_{2}^{2}-\nu \frac{k_{nl}}{3}x_{2}^{3}+k_{c}x_{1}^{2}x_{2},$$

where the oscillator energies are

(83) $$E_{1}=\frac{m_{1}}{2}{\dot{x_{1}}}^{2}+\frac{k_{1}}{2}x_{1}^{2},\;E_{2}=\frac{m_{2}}{2}{\dot{x_{2}}}^{2}+\frac{k_{2}}{2}x_{2}^{2}-\nu \frac{k_{nl}}{3}x_{2}^{3},$$

and potential energy associated with the coupling of the oscillators is defined as

(84) $$E_{12}=k_{c}x_{1}^{2}x_{2}.$$

This yields a total system energy of

(85) $$E=E_{1}+E_{2}+E_{12}.$$

It is assumed that the oscillators are weakly coupled, such that

(86) $$E=E_{1}+E_{2}+E_{12}\approx E_{1}+E_{2}.$$

**Figure 18:**
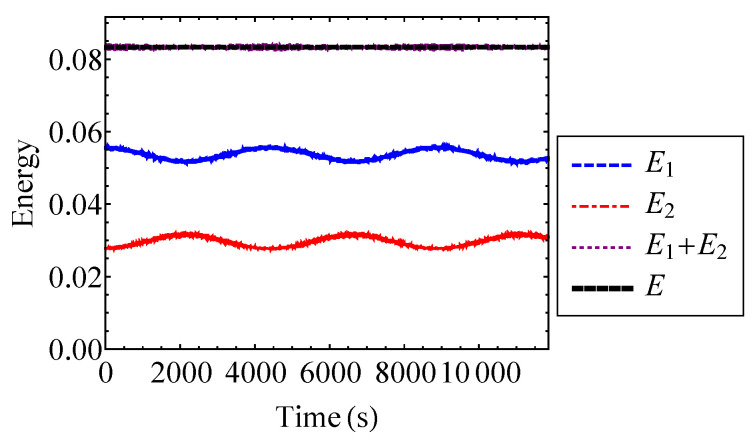
Energies $E_{1}$ and $E_{2}$ of the Henon–Heiles oscillators compared to their sum, $E_{1}+E_{2}$, and the total energy, *E*.

The energies of the Henon–Heiles oscillators are plotted in Figure 18. Figure 18 verifies that the total energy of the system is approximately equal to the sum of the oscillator energies. Thus, the coupling energy, $E_{12}$, is small and the oscillators are weakly coupled. It is shown that the oscillator energies experience a beating phenomenon where low frequency oscillations are interspersed with high frequency noise.

#### 5.2.1. Entropy of the Henon–Heiles Oscillators

The generating function of a linear oscillator is the Laplace transform of the structure function associated with Equation (46). This yields the generating function of the first oscillator:

(87) $$\Phi_{1}\left(s\right)=\frac{2\pi }{\sqrt{km}}\frac{1}{s}$$

Substituting Equation (87) and Equation (79) into the law of composition, Equation (6), the generating function of the Henon–Heiles system is found to the second and fourth order with respect to ν:

(88a) $$\Phi^{\left(2\right)}\left(s\right)=\Phi_{1}\left(s\right)\Phi_{2}^{\left(2\right)}\left(s\right)=\frac{\pi^{2}}{\sqrt{k_{1}k_{2}m_{1}m_{2}}}\left(\frac{4}{s^{2}}+\frac{10k_{nl}^{2}}{3k_{2}^{3}s^{3}}\nu^{2}\right),$$

(88b) $$\Phi^{\left(4\right)}\left(s\right)=\Phi_{1}\left(s\right)\Phi_{2}^{\left(4\right)}\left(s\right)=\frac{\pi^{2}}{\sqrt{k_{1}k_{2}m_{1}m_{2}}}\left(\frac{4}{s^{2}}+\frac{10k_{nl}^{2}}{3k_{2}^{3}s^{3}}\nu^{2}+\frac{385k_{nl}^{4}}{18k_{2}^{6}s^{4}}\nu^{4}\right).$$

Substituting Equation (88) into Equation (8), the value s=σ that minimizes the entropy can be obtained. The second and fourth order series expansions of σ are

(89a) $$\sigma^{\left(2\right)}=\frac{2}{E}+\frac{5k_{nl}^{2}}{12k_{2}^{3}}\nu^{2},$$

(89b) $$\sigma^{\left(4\right)}=\frac{2}{E}+\frac{5k_{nl}^{2}}{12k_{2}^{3}}\nu^{2}+\frac{695Ek_{nl}^{4}}{288k_{2}^{6}}\nu^{4}.$$

The temperature, T=1/σ, of the system is not explicitly calculated in this section. This is to demonstrate that the entropy can be obtained by using σ directly if the system temperature is not a parameter of interest. Substituting Equations (88) and (89) into Equation (7) and setting C=0 yields Khinchin’s entropy for the Henon–Heiles system to the second and fourth order with respect to ν:

(90a) $$H^{\left(2\right)}\left(E\right)=2+\frac{5Ek_{nl}^{2}}{12k_{2}^{3}}\nu^{2}+\ln\left[\frac{E^{2}\pi^{2}}{\sqrt{k_{1}k_{2}m_{1}m_{2}}}\right],$$

(90b) $$H^{\left(4\right)}\left(E\right)=2+\frac{5Ek_{nl}^{2}}{12k_{2}^{3}}\nu^{2}+\frac{695E^{2}k_{nl}^{4}}{288k_{2}^{6}}\nu^{4}+\ln\left[\frac{E^{2}\pi^{2}}{\sqrt{k_{1}k_{2}m_{1}m_{2}}}\left(1-\frac{695E^{2}k_{nl}^{4}}{576k_{2}^{6}}\nu^{4}\right)\right].$$

#### 5.2.2. Mixing Entropy of the Henon–Heiles Oscillators

The hypothetical decoupled entropy of the Henon–Heiles system must be found in order to obtain the mixing entropy. Equation (81) yields the hypothetical decoupled entropy of the nonlinear oscillator with the substitution of the appropriate physical parameters. The hypothetical decoupled entropies of the oscillators are denoted as $H_{1}^{*(2)}\left(E_{1}\right)$ and $H_{2}^{*(2)}\left(E_{2}\right)$ for the second order approximations with respect to ν, and as $H_{1}^{*(4)}\left(E_{1}\right)$ and $H_{2}^{*(4)}\left(E_{2}\right)$ for the fourth order approximations. The total hypothetical decoupled entropy of the system is given to the second and fourth order by

(91) $$H^{*(2)}\left(E_{1},E_{2}\right)=H_{1}^{*(2)}\left(E_{1}\right)+H_{2}^{*(2)}\left(E_{2}\right),\;H^{*(4)}\left(E_{1},E_{2}\right)=H_{1}^{*(4)}\left(E_{1}\right)+H_{2}^{*(4)}\left(E_{2}\right).$$

The mixing entropy is the difference between the actual entropy of the system and the hypothetical decoupled entropy:

(92) $$H_{mix}^{\left(2\right)}=H^{\left(2\right)}\left(E\right)-H^{*(2)}\left(E_{1},E_{2}\right),\;H_{mix}^{\left(4\right)}=H^{\left(4\right)}\left(E\right)-H^{*(4)}\left(E_{1},E_{2}\right).$$

**Figure 19:**
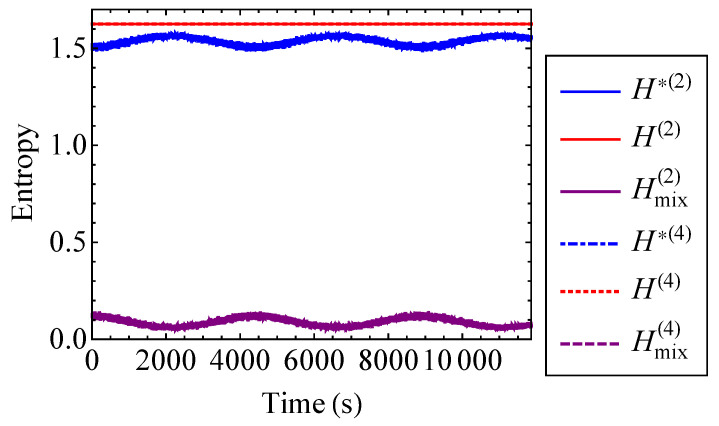
Actual entropy, *H*, total hypothetical decoupled entropy, $H^{*}$, and mixing entropy, $H_{mix}$, of the Henon–Heiles system approximated to the second and fourth order.

The actual entropy, hypothetical decoupled entropy, and mixing entropy of the Henon–Heiles system are plotted to the second and fourth order in Figure 19. The second and fourth order approximations are indistinguishable from one another for each entropy. This confirms that it is sufficient to use the second order approximations to find the entropy of the system. The actual entropies are each a constant value. The oscillations of the hypothetical decoupled entropies are interspersed with high frequency noise and do not reach the value of the actual entropy. Consequently, the mixing entropy is positive. Additionally, since neither oscillator experiences a state of zero energy, the mixing entropy has no singularities. These observations reinforce the notion that the mixing entropy can be a useful tool for understanding the qualitative behavior of systems.

## 6. Conclusions

In this paper, Khinchin’s entropy has been examined for weakly nonlinear oscillators with third and fourth order anharmonic potentials. We have expanded the approach for calculating Khinchin’s entropy linear systems to weakly nonlinear systems using a perturbation method. Defining a dimensionless strength of nonlinearity parameter, ν, allows for the development of such an approach for the case of weak nonlinearity. A perturbation method is used to calculate an explicit equation of phase volume. This calculation is verified against the numerical calculation of phase volume. For Duffing oscillators, it is shown that approximating entropy to the first order with respect to ν is sufficient in the case of weak nonlinearity. For Henon–Heiles oscillators, it is necessary to approximate entropy to the second order with respect to ν to see an improvement over the linear result. The strength of nonlinearity, ν, is limited by the instability of the nonlinear oscillator for the Henon–Heiles system. Higher order approximations are shown to be possible for both the Duffing and Henon–Heiles systems if more accuracy is desired.

It has been shows that a mixing entropy can be obtained for nonlinear systems. For each of the considered system in this paper, the mixing entropy is shown to be nonnegative in the case of weak coupling. As for linear systems, the mixing entropy provides insight into system behavior and represents the increase in system entropy caused by the coupling of oscillators.

Additionally using both the thermodynamic definition of entropy and Khinchin’s entropy, a relationship between vibrational temperature and energy can be defined. This relationship can be used to derive the power flow equation of SEA. This paper opens the path to improve SEA for nonlinear systems in the future work using the entropy concept.

## Abbreviations

The following abbreviations are used in this manuscript:

${\left(.\right)}^{\prime }$	Derivative of (.) with respect to *x*
$\dot{\left(.\right)}$	Time-derivative of (.)
$x_{i}$	*i*-th displacement degree of freedom
$x_{M}$	Maximum displacement over time
$m_{i}$	*i*-th oscillator mass
$k_{i}$	*i*-th spring stiffness
$k_{nl}$	Nonlinear spring stiffness
$k_{c}$	Coupling spring stiffness
*q*	Generalized coordinate
*p*	Generalized velocity
ν	Nonlinearity multiplier
${\left(.\right)}^{\left(i\right)}$	*i*-th order approximation of (.) with respect to ν
${\left(.\right)}^{\left[i\right]}$	*i*-th term in expansion of (.) with respect to ν
*E*	Energy
*T*	Thermodynamic temperature
Σ	System trajectory in phase space
*V*	Equi-energy volume in phase space
Ω	Structure function
Φ	Generating function
*H*	Entropy
$H^{*}$	Decoupled entropy
$H_{mix}$	Mixing entropy
B,C	Scalar constants
σ	Value that minimizes *H*
:=	Equal by definition
q	Generalized coordinate matrix

## Appendix A

Assume ν is small,

(A1) F(x)=∫f(x)dx,

and one can represent F(x) as a polynomial meaning

(A2) $$F\left(x\right)=\int f\left(x\right)dx=\sum a_{i}y^{i}=a_{0}+a_{1}y+a_{2}y^{2}+a_{3}y^{3}+\ldots$$

Under these assumption, we can show

(A3) $$\int_{0}^{c+\nu d}f\left(x\right)dx=\int_{0}^{c}f\left(x\right)dx.$$

The right hand side of Equation (A3) is

(A4) $$\int_{0}^{c}f\left(x\right)dx=F\left(c\right)-F\left(0\right),$$

and the left hand side of Equation (A3) is

(A5) $$\int_{0}^{c+\nu d}f\left(x\right)dx=F\left(c+\nu d\right)-F\left(0\right).$$

(A6) $$\begin{array}{cc} F(c+\nu d) & =\sum a_{i}{\left(c+\nu d\right)}^{i}\\ & =a_{0}+a_{1}\left(c+\nu d\right)+a_{2}{\left(c+\nu d\right)}^{2}+a_{3}{\left(c+\nu d\right)}^{3}+\ldots \\ & =a_{0}+a_{1}c+a_{2}{\left(c\right)}^{2}+a_{3}{\left(c\right)}^{3}+‥‥+O\left(\nu \right)+O\left(\nu^{2}\right)+‥‥\\ & =F(c)+HOT, \end{array}$$

where HOT stand for higher order terms of ν, assuming ν is small. Equation (A6) shows that the right hand side and left hand side of Equation (A3) are equal and therefore,

(A7) $$\int_{0}^{c+\nu d}f\left(x\right)dx=\int_{0}^{c}f\left(x\right)dx.$$
